# A minimal One Health approach? Lessons from the salmon aquaculture crisis in Chile

**DOI:** 10.1080/11287462.2025.2593707

**Published:** 2025-12-04

**Authors:** Bernardo Aguilera, Juan Alberto Lecaros, Jorge Olivares-Pacheco

**Affiliations:** a Facultad de Medicina, Universidad San Sebastián, Santiago, Chile; b Observatorio de Bioética y Derecho, Instituto de Ciencias e Innovación en Medicina, Facultad de Medicina Clínica Alemana Universidad del Desarrollo, Santiago, Chile; c Grupo de Resistencia a los Antimicrobianos en Bacterias Patógenas y Ambientales, GRABPA, Instituto de Biología, Facultad de Ciencias, Pontificia Universidad Católica de Valparaíso, Valparaíso, Chile

**Keywords:** One Health, environmental ethics, salmon aquaculture, multi-institutional collaboration, anthropocentrism, Antimicrobial resistance

## Abstract

This paper explores the application of the One Health approach through an analysis of the response to the 2007 crisis in Chile's salmon aquaculture industry. To evaluate the extent to which the case aligns with a “minimal” One Health framework, we draw on four key dimensions of this framework (methodological, epistemic, ontological, and ethical) and contrast the case with the response to the 2009 Q fever outbreak in the Netherlands. We conclude that the Dutch response to Q fever, characterized by limited institutional collaboration, a narrow disciplinary focus, and an anthropocentric ethical stance, fell short of even a minimal One Health approach. In contrast, the response by the Aquaculture Health Management Program (PGSA) to Chile's salmon aquaculture crisis represents a more integrated approach, involving multisectoral collaboration, interdisciplinary dialogue, and concern for animal and environmental health. While the Chilean case does not fully achieve a strong One Health model, it demonstrates the practical benefits of adopting a minimal One Health perspective, including reduced antibiotic use and improved disease control. The paper concludes that One Health should be understood as a flexible, problem-solving framework, and that clarity regarding its core dimensions is essential for strengthening One Health approaches.

## Introduction: the One Health approach and its main dimensions

The One Health approach has attracted increasing interest as a means to address complex health challenges. From a historical perspective, the One Health approach originated with the notion of “One Medicine” which was introduced by Schwabe in the 1960s to emphasize the interrelationship between human and animal health. This foundational idea has since broadened its scope to include the environment and was given its current name at the “One World, One Health” Conference organized by the Wildlife Conservation Society in 2004 (Pettan-Brewer et al., 2024). One Health was aimed as an operational concept seeking to make a practical and programmatic call to prevent zoonotic diseases and maintain ecosystem integrity for the benefit of humans, their domestic animals, and biodiversity (Lerner & Berg, 2015; Verweij & Bovenkerk, 2016).

In 2021, One Health gained institutional momentum with the creation of the One Health High-Level Expert Panel (OHHLEP), a multidisciplinary advisory panel for the Food and Agriculture Organization of the United Nations, the United Nations Environment Program, the World Health Organization, and the World Organization for Animal Health. This panel was created to provide technical and scientific advice on One Health issues; however, since the Covid-19 pandemic it has focused on zoonotic disease risk reduction and prevention (Mettenleiter et al., 2023).

The OHHLEP has provided the most comprehensive definition of One Health to date (Adisasmito et al., 2022), which is worth citing at length:

*One Health is an integrated, unifying approach that aims to sustainably balance and optimize the health of people, animals and ecosystems. It recognizes that the health of humans, domestic and wild animals, plants, and the wider environment (including ecosystems) are closely linked and interdependent.*

*This approach mobilizes multiple sectors, disciplines and communities at varying levels of society to work together to foster well-being and tackle threats to health and ecosystems, while addressing the collective need for healthy food, water, energy, and air, taking action on climate change, and contributing to sustainable development. (p. 2)*

From an ethical point of view, the concept of One Health reflects the widespread idea that “the health and well-being of humans has been intimately linked to animals and the planet they share” (Evans & Leighton, 2014, *p*. 415). Moreover, its ethical roots can be traced to the origins of bioethics as a discipline. In what has been considered the inaugural reference to the concept of “bioethics”, Jahr (2011 [1927]) claimed that “the fact of a close interrelationship between animal protection and ethics is finally based on the reality that we not only have moral obligations towards fellow humans, but also toward animals, even against plants – in short: toward all forms of life – so that we can speak about ‘Bio-Ethics’” (*p*. 8).

Potter (1970) coined the term “bioethics” nearly half a century later, independently from Jahr but inspired by Aldo Leopold, one of the founding voices in environmental ethics. His work laid the foundations of bioethics as an interdisciplinary approach that reflects on the intersections of ecology, value, and health of human beings on the planet. For Potter, ethics is centered on the bios – life in the world – and aims to connect the health of the Earth, human health, and conservation (Potter & Whitehouse, 1998). Looking at the origins of bioethics can be useful not just to highlight the conceptual links it shares with the One Health approach, but also to show how this approach can be instrumental in “reinvigorating” a bioethics field that has become too narrowly focused on human health (Beever & Whitehouse, 2017).

The advancement of One Health – and the evolution of bioethics alongside it – will ultimately rely on its fruits, however. There have been concerns that strong or “radical” versions of One Health risk being too idealistic to be effectively implemented in real-world contexts (Sironi et al., 2022). In this article we will explore how a “minimal” One Health approach, which is pragmatic but still remains faithful to the foundational aspects of One Health, would be like. To this purpose, we take a two-step strategy. First, assuming that the concept of One Health can be seen as a boundary object that is flexible enough to encompass diverse interpretations (Leboeuf, 2011), we analyze the One Health framework across four dimensions that will allow us to distinguish between minimal and strong approaches to One Health. Secondly, we use the case of the salmon aquaculture crisis in Chile to illustrate what we see as a successful implementation of a minimal One Health approach. We conclude by discussing some of its advantages and shortcomings, as well as some lessons on how to implement a One Health approach.

## Strong and moderate One Health

Drawing on the analysis of Beever and Morar (2019), we will distinguish four key dimensions that “shape the conceptual space of One Health” and that can be used to distinguish between moderate and strong approaches to One Health. Below, we present these key dimensions. To illustrate how mainstream One Health models integrate into this framework, we also assess how the OHHLEP's definition of One Health (presented in the previous section) aligns with each of the dimensions.

• *Methodological dimension: multi-institutional versus interinstitutional*. One Health has traditionally been understood as a practically oriented approach aimed at fostering collaboration between various institutions. However, not all collaboration is equal. In multi-institutional collaborations, each entity typically contributes within its own silo, without deeper integration. In contrast, an interinstitutional approach involves a more organic and purposeful partnership, where institutions codevelop solutions using shared conceptual frameworks. When it comes to OHHLEP's definition, it says that One Health “mobilizes multiple sectors, disciplines and communities” and that it is “an integrated, unifying approach”, which suggests an interinstitutional collaboration.
• *Epistemic dimension: multidisciplinary versus interdisciplinary*. One Health recognizes that the complexity of natural systems often demands not just a collective response but also one in which multiple actors bring different disciplines to the fore, which amounts to multidisciplinary collaboration. Alternatively, stakeholders can pursue a deeper, interdisciplinary collaboration, in which the perspectives from multiple disciplines are combined to create a distinct epistemological perspective. The OHHLEP characterization of One Health is committed to multiple disciplines working together, while its claim that it amounts to an “integrated, unifying approach” is suggestive of interdisciplinarity.
• *Ontological dimension: interconnected versus interdependent*. While all One Health approaches agree that there is a close relationship between the health of humans, animals, plants, and the environment, a debatable issue is whether the nature of the relationship is one of mere interconnectedness, or one of interdependence. Interconnection does not entail interdependence: while the first refers to causal relationships, the second claims that these relationships are co-constitutive and acknowledges the complex dynamics of human–animal–environment interactions (Barrett & Osofsky, 2013). By saying that the “health of humans, domestic and wild animals, plants, and the wider environment … are closely linked and interdependent”, the OHHLEP expresses a commitment with the latter, stronger ontological view.
• *Ethical dimension: moderate versus strong nonanthropocentrism*. Despite its aim at a more relational approach to health problems, in practice One Health approaches have tended to prioritize human interests, treating the interests of nonhuman entities as secondary or instrumental (Van Herten, 2021). From a nonanthropocentric viewpoint, in contrast, the interests of nonhuman entities are considered ethically important in their own right. Although the OHHLEP's aim to “sustainably balance and optimize the health of people, animals and ecosystems” can be interpreted as pointing towards a nonanthropocentric view, it does not specify how to appropriately weigh human versus nonhuman interests.

As we discuss in the next section, traditional public health strategies frequently fall short of meeting even the moderate end of these conceptual dimensions. Following Beever and Morar (2019), we assume that genuine One Health approaches start from the “moderate” side of the four dimensions and move towards “stronger” holistic views such as ecological egalitarianism. According to our assessment, the OHHLEP's definition gets close to the latter view. Despite their theoretical appeal, such strong One Health approaches have been seen as involving “a true epistemological and ethical change that appears neither easy to accomplish nor simple to implement through consistent policies” (Sironi et al., 2022, *p*. 2). We agree with these practical concerns and will contend that what we call a “minimal” One Health approach, i.e. one that fulfills the moderate end of the four domains, stands a stronger chance of succeeding when applied to real-world situations.

## From traditional public health to One Health

The four dimensions mentioned above are important for understanding the conceptual boundaries of One Health and evaluating whether a health intervention reaches what we shall consider a “minimal” side of the spectrum (or “moderate”, in Beever and Morar's terms). Additionally, these dimensions are useful for analyzing One Health approaches since, depending on how each dimension is understood, different methodological, epistemic, ontological, and ethical commitments are made. Differences in these commitments can then lead to disparate ways of dealing with a problem, even from approaches that are supposed to share a One Health philosophy. To further clarify the four dimensions of One Health and its boundaries, we discuss the 2009 Dutch government's intervention for a Q fever outbreak. We will show that despite being regarded as a One Health response by some quarters, this particular intervention does not qualify as a minimal One Health approach.

Q fever is a zoonotic disease caused by the bacterium Coxiella burnetii that can infect various animals and arthropods (WOAH, 2025). It triggered a significant outbreak in the Netherlands between 2007 and 2010, with over 4000 human cases reported, especially concentrated in areas of intensive dairy goat farming (van der Hoek et al., 2012). In humans, this zoonotic disease can cause pneumonia and the main risk factor is proximity to infected goat farms. Despite the various measures implemented to control the disease (abortion in pregnant goats, DNA testing in bulk milk, mandatory vaccination, etc.), these measures have been insufficient.

In 2009, the Dutch government opted for the systematic culling of over 50,000 pregnant sheep and goats on 88 farms, most of which were healthy (Schneeberger et al., 2014). The following year, there was a drastic decrease in reported human cases, from 2355 cases in 2009 to 504 in 2010 (Delsing & Kullberg, 2008). While this decrease is largely attributed to veterinary measures, it can also be related to the increased immunity of the exposed human population (Schneeberger et al., 2014). The Q fever crisis in the Netherlands affected human health, animal welfare, and the environment, so it was appropriate to approach this case from the integrative perspective of One Health (Rahaman et al., 2019). However, as we shall see, the government's initial response follows a traditional public health approach and falls short of a minimum One Health framework, according to its four dimensions.

From a methodological perspective, health was conceived as a “public good” serving human communities and managed by public health specialists, focusing exclusively on the human outbreak of Q fever (Atlas et al., 2010). Besides public health professionals, veterinarians were also involved; however, they had little interaction with one another, and lacked a shared strategic plan. Faced with the risk of an increase in human cases and the economic losses in goat farming, immediate and narrow interests were prioritized instead of balancing and optimizing the health interests of both humans and animals. From a One Health perspective, one would have expected a more substantial multi-institutional collaboration, for example, between health authorities and veterinary professionals from livestock services that oversee agricultural production.

Likewise, from an epistemic perspective, the response was narrowly focused. It was framed from the single disciplinary perspective of public health medicine, which approached the case in a reactive rather than preventive manner, and disregarding the evidence from veterinary professionals, which indicated that the increase in cases before the decision to mass slaughter animals was a result of improved diagnostic and surveillance systems rather than an intensification of the outbreak (Atlas et al., 2010). A proper, One Health response to the Q fever should have recognized the complexity of the ecosystems involved, calling for multi-institutional collaboration and the convergence of multiple disciplines to tackle the problem. For example, the decisions of public health experts should have been better supported by information from veterinary medicine, as well as from other relevant disciplines.

The “weak” methodological and epistemological approach observed in the response to the Q fever outbreak suggests a similarly “weak” ontological perspective within the context of One Health dimensions. In fact, the response suggests that little time was devoted to analyzing the case through the lens of interconnectedness between humans, animals and the environment. Instead, an “independent” perspective prevailed, in which the problem was understood as consisting of distinct and separate units that can mutually affect each other. More specifically, the Dutch government prioritized human health and the economy over the other parts, resulting in the culling of 50,000 goats. This approach also reflects how the interests of humans overwhelmingly took precedence over those of the animals.

This brings us to the ethical dimension of One Health. In the case of Q fever, an anthropocentric perspective was evident, leading to the culling of thousands of nonhuman animals for the sake of human health. It is worth recalling that even though One Health was initially focused on zoonotic diseases (such as the Q fever), it has always acknowledged the moral importance of animal health and well-being in relation to their natural environment (Meijboom & Nieuwland, 2018). In the present case, concern for the environment was virtually nonexistent. This neglect of the environment is not uncommon even in alleged One Health approaches (Van Herten, 2021), even though emerging environmental challenges and concerns, such as climate change, have pushed One Health approaches towards taking the environmental component more seriously.

In sum, the 2009 Dutch government's response to the Q fever outbreak fits within a conventional public health framework, which, when seen from the four dimensions of One Health, does not qualify to even a minimal conception of One Health. We turn to a novel case study, the response to the salmon aquaculture crisis in Chile in 2007, which we analyze in light of these four dimensions. Our aim is to show that, in contrast with the Q fever case, the salmon aquaculture industry case fulfills the methodological, epistemic, ontological, and ethical conditions for being considered a One Health approach, under our minimal definition, but leaves considerable room for working towards a stronger One Health response.

## The salmon aquaculture crisis in Chile: a case study

The salmon aquaculture industry in Chile has been steadily growing since its beginnings in the 1970s and has become the world's second-largest exporter of salmon. However, this growth has brought important challenges related to the use of antimicrobials, environmental sustainability, and public health (Miranda et al., 2018; Quiñones et al., 2019). The industry's development began with experimental initiatives in the 1970s, and in the 1980s, the first floating cages were established in the Los Lagos Region, marking the start of exports to Japan and the United States. During the 1990s, the industry experienced exponential growth, consolidating Chile as a global leader in this industry (Salmon Chile, 2024).

The rapid expansion of the salmon aquaculture industry, coupled with inadequate regulation, led to significant fish health problems. Among these, the Salmon Rickettsial Syndrome (SRS), caused by the bacterium *Piscirickettsia salmonis* (PS), and the Infectious Salmon Anemia (ISA) virus crisis between 2007 and 2010 stand out (Bachmann-Vargas et al., 2021; Schober et al., 2023). This latter outbreak had a devastating impact, with economic losses exceeding two billion dollars. The event revealed serious shortcomings in biosecurity practices and pathogen management, and led to an increase in the use of antimicrobials as a stopgap measure (Asche et al., 2009; Mardones et al., 2011).

This massive use of antibiotics had detrimental effects on aquatic ecosystems, however. The presence of these compounds in the water column and marine sediments significantly altered the microbiomes associated with salmon production (Miranda et al., 2018; Ortiz-Severín et al., 2024). Furthermore, it contributed to considerable eutrophication events on the seabed. The latter refers to high accumulations of oxygen and carbon, chemically saturating the environment and negatively affecting the development of some species (Yang et al., 2017). Specifically, one of the consequences related to eutrophic events is the massive occurrence of harmful algal blooms, which impact the aquatic food web by altering its dynamics (Quiñones et al., 2019). These detrimental effects can be exacerbated by the fact that some antibiotics are nonbiodegradable, can remain active for years in the aquatic environment, and may induce antimicrobial resistance in surrounding microorganisms (Bhatt & Chatterjee, 2022; Yang & Wu, 2023).

This scenario raised many questions about human health, salmon health, and the aquatic environment: Could the pathogens affecting salmon also impact humans? How might antibiotic use in fish influence human health? How does salmon production affect the environment to the extent of jeopardizing the industry itself? To address these concerns, the National Fisheries Service (SERNAPESCA) created in 2015 the Aquaculture Health Management Program (PGSA). One of the authors of this article (JO), an academic biologist, was a participant of this program from 2015 to 2020, and this case study will partly draw on this experience.

The PGSA gathered representatives from different sectors, including governmental agencies (SERNAPESCA itself), the aquaculture industry, and the academic sector, including universities and research centers. With nearly 15 million dollars in funding, the program supported 47 projects aimed at addressing key challenges in the Chilean salmon aquaculture sector, including disease control, antimicrobial practices, and sustainable production. The academic sector proposed working under a One Health paradigm early in the process, and soon, the PGSA as a whole adopted a One Health approach.

From the outset, the program was built upon tripartite working groups that met in Chilean cities such as Valparaíso, Santiago, Concepción, and Puerto Montt. These meetings fostered an unprecedented level of dialogue and cooperation among stakeholders. There were also symposia in Puerto Montt (south of Chile), where project results were shared, and strategies were debated, and concrete actions were jointly defined and later adopted by the industry. One of the most remarkable outcomes was the successful coordination of research teams from different disciplines – and even traditionally competing groups – who joined efforts under a collaborative framework to address shared problems.

The PGSA focused on four key goals:

1. Enhancing the knowledge of salmonids' genetic and immunological traits.
2. Deepening the understanding of the main pathogens impacting the industry (including Piscirickettsia salmonis, the ISA virus, and the sea louse Caligus rogercresseyi).
3. Regulating the use of antimicrobials.
4. Assessing the environmental impact of salmon aquaculture.

Regarding the goal of regulating antimicrobials, the PGSA led to the establishment of the Antimicrobial Use Optimization Program (PROA-Salmon), aimed at promoting the rational use of these drugs and strengthening three key aspects: (a) ensuring food safety by maintaining low concentrations of antibiotics in salmon meat, thereby ensuring its safe consumption by people; (b) preventing the emergence of antimicrobial resistance in pathogenic bacteria affecting salmon; and (c) preserving an environment with low antibiotic loads, thus minimizing the selection of resistant bacteria in areas surrounding fish farms.

Finally, the fourth goal of the PGSA, which was to assess the environmental impact of salmon aquaculture, led to various preventive and corrective measures aimed at a more sustainable salmon aquaculture industry: (a) zoning and clustering of aquaculture centers, which allows establishing management zones and isolation areas to prevent the spread of diseases; (b) the implementation of sanitary surveillance plans through constant monitoring of fish health with regular sampling and laboratory analysis for early detection of pathogens; (c) the adoption of biosecurity measures, such as restricting the movement of fish and materials between farming centers, the use of physical barriers, and strict disinfection protocols to prevent the introduction and spread of diseases; (d) the development of contingency plans that include specific protocols to address disease outbreaks, including sanitary culling, quarantine, vaccination campaigns (for PS and ISA) and the safe disposal of biological waste; (e) seabed cleaning plans, and (f) reducing the stocking density of fish in farming centers. It is also worth mentioning that the PGSA laid down enforcement and compliance regulations that included regular inspections and audits by national authorities.

**Figure 1.**
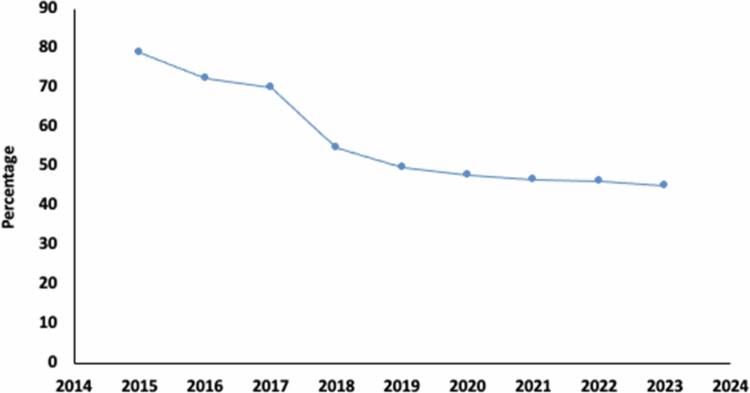
The percentage of mortality due to infectious causes attributed to SRS in Atlantic salmon, based on data from Sernapesca. It shows a decreasing trend in the relative importance of SRS observed from 2015 to 2023. Source: SERNAPESCA (2025).

We believe that the PGSA is an example of how collaboration between different sectors and disciplines can lead to effective and sustainable solutions. The program achieved tangible outcomes. First, salmon diseases were controlled. The mortality rate attributed to SRS as an infectious disease dropped from 78.9% in 2015 to 45% in 2023 in the Atlantic salmon (Figure 1). Considering the initially high mortality rate, the sustained implementation of the program's multifaceted strategies is likely to lead to further reductions in mortality.

**Figure 2.**
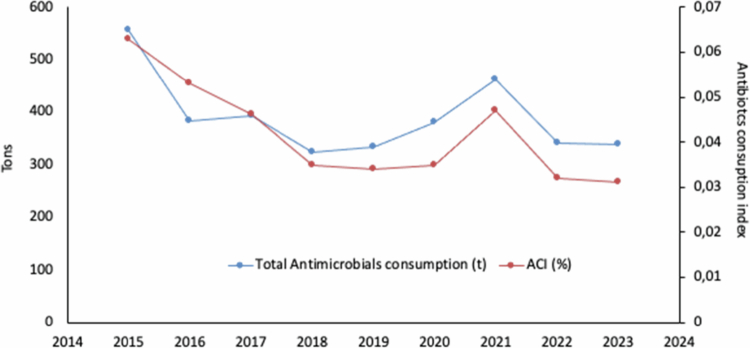
Blue line: Total tons of antibiotics used. Orange line: Antibiotics consumption index ACI (quantifies the amount of antibiotics used relative to the biomass produced and is expressed in grams of active ingredient per ton of biomass (g/t)). Source: SERNAPESCA (2025).

Second, the use of antibiotics was also reduced by 51.8% between 2015 and 2023, while total consumption fell by 39.1% during the same period (Figure 2).

Third, the program led to the development of more robust fry better equipped to resist diseases in seawater, and the implementation of functional feeds (feeds designed to have positive effects on the immune system or disease resistance in farmed fish) that improved fish health and reduced the need for antimicrobials.

Finally, as previously noted, there was a consensus about adopting a One Health approach, which resulted in discussions about how to address concerns about animals and the environment. By bringing together expertise from microbiology, marine biology, ecology, veterinary medicine, animal production, pharmacology, oceanography, and engineering, the program embodied a systemic, science-based approach that addressed the complexity of aquaculture in a holistic way. This experience not only contributed to the advancement of the Chilean aquaculture sector but also offered a blueprint for the design of future One Health policies rooted in interdisciplinary cooperation and scientific evidence.

Despite these achievements, it is important to recognize that major challenges persist, in particular to restore the biodiversity affected by past practices and improving fish welfare. Some measures oriented to address these challenges include: (1) the reduction of cage densities, giving salmon more space to swim and move, as well as mitigating problems related to oxygen deficiency in farming areas; (2) the implementation of sustainable feeding conditions for fish, which can include using natural product-based feeds that act as immunostimulants, enhancing fish health and thereby reducing the use of antibiotics; and (3) implementing safe, appropriate slaughter protocols aimed at promoting animal welfare.

## Discussion of the case from a One Health approach

In the case study we see how the salmon aquaculture industry in Chile was challenged by complex biological and environmental factors, and how this challenge was dealt with a (self-proclaimed) One Health strategy. The case tells a story of success, at least in terms of combating salmon pathogens with less antibiotics and recovering industry production. We will start this discussion by analyzing the case based on the four dimensions of One Health. Our conclusion will be that the case of Chile's salmon aquaculture crisis can be qualified as a One Health response, albeit a minimal one, that has considerable room for further advancing in terms of the “strength” of the One Health approach. We also discuss how feasible that might be. While we recognize that the ethics and sustainability of salmon aquaculture has already been debated in the academic literature, particularly with regard to its social impacts, animal welfare, conservation of other species, and marine ecological balances, it will not be the main focus of this article (Bailey, 2014; Martin et al., 2021; Olesen et al., 2011).

In the case of Chile’s salmon aquaculture crisis, we see a multi-institutional and multidisciplinary response that takes into account the complexity of the challenge ahead. In contrast with the Dutch Q fever case, in which the response was limited to public health experts and institutions, in the Chilean salmon aquaculture case we find a collective effort in which governmental, industry, and academic sectors worked together during an extensive period. A lesson from this experience is that political will (in this case, represented by the pivotal role of SERNAPESCA) was key to provide support and continuity to this forum of dialog between different sectors. Despite the fact that there were effective mechanisms of coordination between institutions (e.g. periodic SERNAPESCA memorandums), we believe that, in terms of its methodology, it is fair to qualify the case as an instance of multi-institutional – and not interinstitutional – collaboration, since the PGSA response remained confined to the scope of each institution and did not move beyond their boundaries.

The multi-institutional conformation of the PGSA suggests that multiple disciplines were also involved. In fact, the program gathered biologists, veterinarians, public health experts, oceanographers, pharmacologists, engineers, business leaders, and government officials, among others. There was therefore an acknowledgment that salmon diseases are embedded in a complex and interconnected system that involves not just animals and their aquatic environment, but also human beings within social and economic structures. A lesson from this case is that the early adoption of the One Health approach, driven by participants with background in academia, provided a platform for dialog and productive work among multiple disciplines and stimulated work beyond traditional disciplinary boundaries. We refrain, however, from qualifying the work of the PGSA as interdisciplinary for two reasons. One is that even though the One Health approach was relevant in framing the beginning of the collective effort, the program lacked an integrated and consistent conceptual approach; rather, it remained applied and industry-centered. The second reason is that most reports from this program were issued independently by each institution and framed in terms of their respective disciplines.

As mentioned, the multidisciplinary approach to the salmon aquaculture crisis stemmed from an acknowledgment of its complexity. Reflecting trends in the emerging literature, there was a recognition that sustainable solutions in salmon aquaculture require taking into account “its environmental, social and economic aspects – and the trade-offs among these.” (Bailey, 2014, *p*. 23). This contrasts with mono-causal approaches, such as the one discussed in the Dutch Q fever case, or the approach of the Chilean industry to salmon diseases that preceded the PGSA (which was basically to increase the use of antibiotics). From an epistemic perspective, however, we believe that the overall understanding of the PGSA to this issue was in terms of interconnectedness rather than interdependence. As mentioned, the reporting of the PGSA remained parceled and confined to disciplinary boundaries, and even though during internal meetings and public seminars there was interest in, and discussions about, how changes in one part of the ecosystem can have widespread consequences, the decisions that were finally adopted did not go that far. For example, potential downstream effects to human health were virtually ignored in the reports and conclusions of the PGSA.

The main factors behind the creation of the PGSA were the needs of the salmon aquaculture industry and therefore one can presume an anthropocentric motivation. However, the involvement of multiple disciplines and the mentioned recognition of the complex causal structures of ecosystems led to concern towards the health and well-being of nonhuman entities. We can see actions that were aimed at improving the well-being of salmon (e.g. isolation of aquaculture centers and optimization of protocols to address disease outbreaks) and at taking care of the environment (e.g. removal of inorganic waste from the seabed and reduction of fish density in farming centers to prevent eutrophication processes or decreases in oxygen concentration in the water body, which could lead to the death of other species.).

One might argue that these actions were motivated by a merely instrumental concern for nonhuman entities and therefore do not represent a genuine nonanthropocentric viewpoint. Even though there is some truth in this claim, we believe that some of the actions taken in this respect were promoted by actors who were committed to a One Health approach and were inclined to attribute intrinsic moral value to animals and the environment. In the course of the PGSA discussions, for example, biologists often raised the issue of salmon well-being, and some meetings included the participation of environmental advocacy groups such as Oceana Chile, which at least suggests that nonanthropocentric views were present. Again, all this is at variance with traditional approaches such as the in the Dutch Q fever case, where decisions reflect an almost exclusively anthropocentric viewpoint.

In sum, we believe it is fair to say that the case of the salmon aquaculture industry in Chile constitutes a One Health response which, albeit minimal, signs a departure from traditional public health approaches lacking some or all of the hallmarks of One Health. It is important to stress, however, the minimal (some might say, tokenistic) character of the One Health approach here. To their credit, some stakeholders involved in this case – especially those from academic institutions – often recognized that there are challenges ahead in terms of determining downstream effects of their policies and taking care of the environment and one can see openness for a stronger One Health approach. However, during the program, a more realistic, instrumental, and pragmatic strategy was adopted aimed at making the salmon aquaculture companies sustainable by reducing the impact on animal health and aquatic ecosystems, as a first step that can lead to a more integrative and unified approach to One Health.

We find similar positive narratives in the literature. For example, in a recent review, Avendaño-Herrera et al. (2023) praise the results of Chilean policies (e.g. in reducing the use of antimicrobials) that they describe as “aligned with the One Health” approach. The definition of One Health adopted by the authors says that it is “an approach to designing and implementing programs, policies, legislation and research in which multiple sectors communicate and work together to achieve better public health outcomes”. Even though this definition suggests the participation of multiple institutions and disciplines, it falls short of addressing the ontological and ethical dimensions of One Health.

This rather narrow understanding of One Health is representative of Avendaño-Herrera et al.'s assessment of the case of the Chilean salmon aquaculture industry. For example, their calls for “mitigation plans for the environments and ecosystems in which the antimicrobial is applied” and for an “environmentally friendly and sustainable” industry suggest an instrumental and rather peripheral preoccupation for the environment (Avendaño-Herrera et al., 2023). Overall, we believe that the author's take of the One Health approach reinforces our appraisal of the PGSA response as rather limited in terms of the dimensions of One Health.

In the way of contrast, consider the work of Cabello et al. (2023), who claim that framing the case of the salmon aquaculture industry in Chile as a success story can be misleading. They claim that the PGSA response to the challenge was strongly influenced by industry interests and has been short of representing a robust dialog among all stakeholders. They also highlight potential unexpected or downstream effects of the high use of antimicrobials. For example, the potential for *de novo* emergence of antimicrobial resistance genes in bacteria subjected to high antibiotic concentrations, and the ability of bacteria of the aquatic environment to spread their antimicrobial resistance genes with terrestrial animals and microorganisms lead to a loss of antimicrobial efficacy that can affect humans and other animals. This shows that for proponents of a stronger One Health approach the PGSA response can be seen as shallow and short-sighted.

To repeat, our own view is that the case of the salmon aquaculture industry in Chile does represent a One Health approach, albeit in its minimal form, and that its successful results are a product of having adopted this perspective. In this sense, one can remain within the conceptual space of One Health without having to settle the issue of whether a certain intervention is multi‐ or interinstitutional, multi- or interdisciplinary, committed to interconnectedness or interdependence, or fully nonanthropocentric. Furthermore, since its origins One Health has been a practically oriented approach, aimed at solving health problems. Therefore, we believe that rigid, uncompromising views on the One Health approach can be inconsequential. As the PGSA response shows, One Health can be pivotal for the generation of a multisectoral response, that fueled by political will, can deal with complex health challenges and deliver tangible results.

A further lesson from this case is that the dimensions of One Health stand in a reciprocal and hierarchical relationship. How one approaches the methodological dimension, influences how the epistemic, and then the ontological, and then the ethical dimensions will be understood. If, for example, a health problem is dealt with by a single institution with little enthusiasm for multisectoral collaboration, the response will likely remain outside the scope of One Health. In contrast, when from the beginning there is a commitment for multisectoral, inclusive, and coordinated response, there are high chances that at least multidisciplinarity, interconnectedness and nonanthropocentrism will be under the table. This reinforces our view that it is important to have clarity about the different dimensions of One Health since decisions regarding how to approach them can have important practical and normative implications.

## Conclusions

In contrast with traditional responses to health challenges (such as the Dutch 2009 response to the Q fever), the case of Chile's salmon aquaculture crisis exposed in this article represents a meaningful, though “minimal”, application of the One Health approach. While the response to the Chilean salmon aquaculture case, led by the PGSA initiative, succeeded in assembling a multi-institutional response to a complex challenge, as well as in reducing antibiotic use and stabilizing salmon production, it fell short of achieving full interinstitutional and interdisciplinary integration. The PGSA response leaned more toward recognizing interconnectedness than toward embracing true interdependence, and its ethical stance remained largely anthropocentric, despite some efforts to consider animal welfare and environmental health. Nonetheless, the case highlights the practical value of One Health as a flexible, problem-solving framework. It underscores the importance of understanding and clarifying its methodological, epistemic, ontological, and ethical dimensions, which are deeply intertwined. As such, the Chilean experience offers valuable lessons for strengthening future One Health initiatives and advancing toward more holistic and inclusive health governance.
